# Modern Sociology

**Published:** 1899-06-03

**Authors:** 


### Modern Sociology.

### PREGNANCY AND FACTORY WORK.

Our Factory Acts ordain that no woman shall be employed in a factory within four weeks after her confinement, which is so far good. But they make no limitation of the circumstances under which a woman may work before her confinement. Yet this is not less important. A woman who exerts herself too soon and too severely after childbirth may injure herself; but a woman who works too hard before the birth of her child may very easily injure the child. From the point of view of the State this is perhaps even more serious. The State can hardly control adults, even if their mode of life is foolish and likely to injure their health; each is supposed to be able to look after himself, and our nation has never yet looked kindly on plans to make people virtuous or happy by Act of Parliament. But the State is bound to look after the welfare of its future citizens. It is for this reason that the work of children is so carefully regulated, even where it is not wholly forbidden. Free agents may make their own bargains, be these foolish or wise, hard or the reverse; but it is the State's duty to look after the welfare of those who are not free in the making of the bargain. Yet that the rights of the unborn are sacrificed every day in all classes of society is hardly to be denied, and among factory workers it is the exception, rather than the rule, for a woman to give up work when she has the prospect of becoming a mother.

It may seem strange that the Factory Acts do not touch this important point, and in some countries they do; but it is doubtful if such provisions as those which ordain that a woman shall not work within a certain time before her confinement could be enforced. As a matter of fact it would depend on the woman herself, as it does now, to say at what date she should stop work. It is impossible for an employer to be saddled with the responsibility of saying when any of his women workers should leave, therefore he cannot be held responsible if one or another works too long; and when one considers the chances of premature births it becomes obvious that no employer can be held liable for injury to a woman through her working till too near her confinement. But Factory Acts operate through the employer. It is he who is, at least apparently, to be coerced by them; it is he who is punished if they are broken. No doubt they are in many cases a real protection to the employer, who can fall back on them when a worker wants to do things which are forbidden. He dare not employ children, young persons, or women under other conditions than those laid down by the law, and this saves him many a time from a position that might, if he acted without such sanction, be regarded as ungracious, if not harsh. But the case of the pregnant woman is beyond the employer's power to tackle.

It is, of course, possible to forbid all married women to work in factories, but this would bear with unreasonable hardness on women who were not pregnant. True it is that it is better for married women to confine their industry entirely within the walls of their own homes, as a general rule, but that is a matter for social feeling, not for legal enactment. If it could be made a conventional propriety for married women to ive up work at their marriage you would soon see even those who could ill afford to lose the wages refuse to go out; but that day is a long way off yet, and the tendency of the age is rather opposed to it than otherwise. Besides, any regulation as to the work of married women would do nothing for illegitimate children, who, having other disadvantages to contend with in life, have rather a greater need than those born in wedlock of a good constitution, and among factory workers illegitimacy is too common. Whether this is due in any way to the conditions of factory life is not certain. Some observers think that with men and women working together a loose tone of talk and manners is engendered, which tends to laxity of morals. This may be; but we are rather inclined to set down the cause of the unchastity of factory girls to the fact of their economic independence. The factory girl maintains her own child. She earns, perhaps, as much as many of the male workers; the child is no more of a burden and disadvantage to her than to the married woman who works next her. Being economically independent of public opinion, she can face the world, and therefore public opinion is not so hard on her as on those cases which appeal to it *ad misericordiam*. But, in any case, all possible Factory Acts leave her untouched.

What is necessary to prevent pre-natal injury to children is an increase in knowledge, and in the sense of parental responsibility among the industrial population themselves. To this end health lectures and classes may be found useful, and a well-trained and conscientious district nurse should be almost a missionary. But it is a matter in which the State can do nothing. Reform must come from the people themselves. A recent writer, himself descended from working people, declares that the women who work in the factories try to procure miscarriage by such crude and dangerous methods as jumping off tables and the like, as well as by taking all the advertised nostrums to that end. To them a child is only a hindrance, an unwelcome result of married life, another mouth to fill. Now, while this view predominates it is impossible for any external agency, legal or other, to prevent the murder of the unborn, and enfeebled constitutions to such as manage to see the light. Till expectant parents have a higher moral sense all extension of Factory Acts would be futile. Of course, the plea is put forth that women go to work in the factories because wages are so small that the man's earnings will not keep the household. Where this excuse is well founded it usually turns out to be a case in which two young people have married at a ridiculously early age, and without either provision for the future or much hope of better prospects. Poverty is bound to result in any class from such foolish lack of forethought; and it would be better if the young men and women of the working class would wait for a few years before taking upon themselves the responsibilities of matrimony. There would then be less need for the wife to go out to work, less chance of the possible children being, wilfully or witlessly, sacrificed. But, as a matter of fact, a girl who has been accustomed to the stir and bustle and to the companionship of factory life does not care to settle down to unalleviated domesticity. She feels dull and lonely, and wants to go back to work, even though there is not the excuse of necessity, and therein lies, perhaps, the root of all the trouble.

